# Long noncoding RNA LINC01124 activates hepatocellular carcinoma cell proliferation, migration, and invasion by absorbing microRNA-1247-5p and overexpressing FOXO3

**DOI:** 10.32604/or.2022.03550

**Published:** 2022-08-01

**Authors:** LEI SUN, YUE ZHANG, YUQIN YAO, HONGLIN DU, YUEHUA ZHANG, AIPING FANG

**Affiliations:** 1 Department of Geriatric Medicine, West China School of Public Health and West China Fourth Hospital, Sichuan University, Chengdu, 610041, China; 2 State Key Laboratory of Biotherapy/Collaborative Innovation Center for Biotherapy, West China Hospital, Sichuan University, Chengdu, 610041, China; 3 Department of Hepatobiliary and Pancreas Surgery, Second Clinical Medical College of Jinan University (Shenzhen People’s Hospital), Shenzhen, China; 4 Respiratory Disease Research Center, West China School of Public Health and West China Fourth Hospital, Sichuan University, Chengdu, 610041, China

**Keywords:** HCC, Competing endogenous RNA theory, Anticancer treatments

## Abstract

Long intergenic non-protein coding RNA 1124 (LINC01124) has been identified as an important regulator of non-small-cell lung cancer. However, the expression and detailed role of LINC01124 in hepatocellular carcinoma (HCC) remain unestablished to date. Therefore, this study aimed to elucidate the role of LINC01124 in the aggressiveness of HCC cells and identify the underlying regulatory mechanism. Quantitative reverse transcriptase-polymerase chain reaction was performed to measure the expression of LINC01124 in HCC. Cell Counting Kit-8 assay, Transwell cell migration and invasion assays, and a xenograft tumor model were used to investigate the function of LINC01124 in HCC cells, and bioinformatics analysis, RNA immunoprecipitation, luciferase reporter assay, and rescue experiments were used to elucidate the underlying mechanisms. Herein, LINC01124 overexpression was confirmed in HCC tissues as well as cell lines. Further, the downregulation of LINC01124 decreased HCC cell proliferation, migration, and invasion *in vitro*, whereas the upregulation of LINC01124 triggered the opposite results. Additionally, LINC01124 ablation impaired tumor growth *in vivo*. Mechanistic analyses revealed that LINC01124 functions as a competing endogenous RNA to sponge microRNA-1247-5p (miR-1247-5p) in HCC cells. Moreover, forkhead box O3 (FOXO3) was identified as a direct target of miR-1247-5p. FOXO3 was positively regulated by LINC01124 in HCC cells through the sequestration of miR-1247-5p. Finally, rescue assays revealed that the inhibition of miR-1247-5p or overexpression of FOXO3 reversed the effects of LINC01124 silencing on the HCC cell malignant phenotype. In summary, LINC01124 plays a tumor-promoting role in HCC by regulating the miR-1247-5p–FOXO3 axis. The LINC01124–miR-1247-5p–FOXO3 pathway may provide a foundation for the identification of alternative therapies for HCC.

## Introduction

Liver cancer is one of the most prevalent human cancers and the third leading cause of death globally [1]. Approximately 800,000 cases of liver cancer are newly diagnosed and over 780,000 cancer-related deaths are reported annually worldwide [1]. Notably, hepatocellular carcinoma (HCC) represents approximately 75%–85% of all liver cancer cases [2]. Increased understanding of the biology of these tumors and the development of novel diagnostic and targeted therapeutic approaches has improved HCC treatment in recent years; however, the clinical efficacy and long-term overall survival rates remain highly unsatisfactory [3]. A combination of high degree of malignancy, unlimited growth, metastasis, antiapoptotic phenotype, and recurrence leads to poor prognosis of patients with HCC [4]. Therefore, it is important to fully clarify the molecular mechanism underlying HCC pathogenesis and progression to help identify effective diagnostic markers and improved treatment strategies.

Long noncoding RNAs (lncRNAs) are a group of RNA transcripts with lengths of 200–1,000 nucleotides [5]. Because lncRNAs do not encode proteins, they were originally considered “transcriptional noise” [6]. However, lncRNAs have recently been recognized to exert an epigenetic function by modulating both transcriptional and posttranscriptional gene expression in human cells [7]. Differentially expressed lncRNAs are associated with carcinogenesis and cancer progression [8–11]. The aberrant expression of lncRNAs in HCC has been widely reported in recent years [12]. lncRNAs, which act as tumor-suppressing or tumor-promoting factors in HCC, have been implicated in the regulation of numerous aggressive oncogenic processes [13–15].

MicroRNAs (miRNAs) are approximately 17–24-nucleotides-long RNA molecules that are not translated into proteins [16]. miRNAs directly bind to the 3′-untranslated region of their target genes through complementary base pairing and exert a negative regulatory effect on target gene expression, resulting in mRNA degradation and/or translation inhibition [17,18] MiRNAs are important gene expression regulators that contribute to the tumorigenesis and progression of HCC by influencing the malignant phenotype of tumors [19,20]. Recent evidence has revealed a crosslink between miRNAs and lncRNAs, known as the competing endogenous RNA (ceRNA) theory [21–23]. LncRNAs function as ceRNAs or molecular sponges that sequester miRNAs, thereby reversing the miRNA-mediated inhibition of their target mRNAs [24,25]. Therefore, an in-depth study of the detailed roles of lncRNAs and miRNAs in HCC can help identify new diagnostic markers and therapeutic targets.

Aberrant expression of LINC01124 has emerged as an important regulator in non-small cell lung cancer [26]. However, the biological function of LINC01124 in HCC remains unknown. Through TCGA dataset, we found that LINC01124 ranks 53^rd^ among all overexpressed lncRNAs in HCC. Therefore, LINC01124 was selected for further experiment. In this study, we measured LINC01124 expression in HCC as well as elucidated the regulatory effects of LINC01124 on HCC cells and identified the putative mechanisms underlying the action of LINC01124 as an oncogenic lncRNA through functional experiments.

## Materials and Methods

### Tissue samples

This study was approved by the Ethics Committee of the West China Hospital of Public Health. All participants were informed about the details of this study and they provided written informed consent prior to participation. A total of 54 HCC tissues and adjacent normal tissues were collected from patients at the Affiliated Hospital of Chengde Medical University. Adjacent normal tissues were obtained at least 2 cm away from HCC tissues. The inclusion criteria were as follows: (i) patients diagnosed as HCC; (ii) was not treated with radiotherapy or chemotherapy prior to surgery; and (iii) agreed to take part in the research. The exclusion criteria were as follows: (i) patients with any other clinical disorders; (ii) patients who had been treated with radiotherapy or chemotherapy before surgery; and (iii) did not agree to take part in the research. The tissues were immediately frozen in liquid nitrogen until use.

### Cell lines

Transformed human liver epithelial-2 (THLE-2) cells were purchased from the American Type Culture Collection (ATCC; Manassas, VA, USA) and cultured in bronchial epithelial basal medium (BEGM^™^; Clonetics Corporation, Walkersville, MD, USA). RPMI-1640 (Gibco; Thermo Fisher Scientific, Inc., Waltham, MA, USA) supplemented with 10% heat-inactivated fetal bovine serum (FBS; Gibco; Thermo Fisher Scientific, Inc.) was used for culturing the HCC cell line SNU-398 (ATCC).

The HCC cell lines SNU-182, Hep3B, and Huh-7 were provided by the Cell Bank of the Chinese Academy of Sciences (Shanghai, China). The Hep3B cell line was maintained on minimal essential media (Gibco; Thermo Fisher Scientific, Inc.) containing 10% FBS, 1% GlutaMAX, 1% nonessential amino acids, 1% sodium pyruvate 100 mM solution, 100 U/mL penicillin, and 100 ng/mL streptomycin. The Huh-7 cell line was cultured in DMEM (Gibco; Thermo Fisher Scientific, Inc.) added with 10% FBS, 1% GlutaMAX, and 1% nonessential amino acids. The culturing conditions for the SNU-182 cell line were the same as those for HuH7, except that the RPMI-1640 (Gibco; Thermo Fisher Scientific, Inc.) was used as basal medium. Moreover, 1% penicillin–streptomycin mixture was added to all culture media. All cells were maintained at 37°C in a humidified incubator equipped with 5% CO_2_.

### Transfection experiments

Small interfering RNAs (siRNAs) targeting LINC01124 (si-LINC01124), FOXO3 (si-FOXO3), and negative control (NC) siRNA (si-NC) were designed and synthesized by GenePharma (Shanghai, China). LINC01124 and FOXO3 were overexpressed in cells by transfecting the LINC01124 overexpression plasmid pcDNA3.1–LINC01124 and FOXO3 overexpression plasmid pcDNA3.1–FOXO3 (Sangon Biotech Co., Ltd.; Shanghai, China), respectively. The miR-1247-5p mimic and inhibitor (RiboBio; Guangzhou, China) were used to increase and decrease endogenous miR-1247-5p expression, respectively. Furthermore, the NC miRNA mimic (NC mimic) and NC inhibitor functioned as controls for the miR-1247-5p mimic and inhibitor, respectively. For the transfection experiments, cells were seeded into 24-well plates 1 day prior to transfection. Lipofectamine® 2000 (Invitrogen; Thermo Fisher Scientific, Inc., Waltham, MA, USA) was used for transfection according to the manufacturer's protocol.

### Quantitative reverse transcriptase-polymerase chain reaction

Total RNA isolation was performed using TRIzol reagent (Invitrogen; Thermo Fisher Scientific, Inc.). A NanoDrop^™^ 2000 spectrophotometer (Thermo Fisher Scientific, Inc.) was used to assess RNA quality. For miR-1247-5p quantitation, purified RNA was reverse-transcribed into cDNA using the miScript Reverse Transcription Kit (Qiagen GmbH, Hilden, Germany), and quantitative PCR was performed using the miScript SYBR Green PCR kit (Qiagen GmbH). The expression of miR-1247-5p was normalized to that of U6 small nuclear RNA. To analyze the expression of LINC01124 and FOXO3, reverse transcription using the PrimeScript^™^ RT reagent kit with gDNA Eraser and quantitative PCR with TB Green® Premix Ex Taq^™^ II (Takara, Dalian, China) were performed, respectively. Finally, the LINC01124 and FOXO3 expressions were normalized to GAPDH expression. Relative gene expression was calculated using the 2^−ΔΔCq^ method.

### Cell Counting Kit-8 assay

The Cell Counting Kit-8 (CCK-8) assay (Dojindo, Tokyo, Japan) was used to measure HCC cell proliferation. Transfected HCC cells were collected and seeded (1 × 10^3^ cells/well) into 96-well plates. The CCK-8 reagent (10 µl) was added on days 0, 1, 2, and 3 after seeding, and the cells were incubated for an additional 2 h at 37°C. The absorbance of each well at 450 nm was measured using a microplate reader (Bio Rad Laboratories, Inc., Hercules, CA, USA). The assay was performed with five replicates and repeated thrice.

### Transwell migration and invasion assays

Transwell filters (8 μm; BD Biosciences, Franklin Lakes, NJ, USA) were used for migration and invasion assays. For the migration assay, 5 × 10^4^ cells were suspended in 100 μl of FBS-free culture medium and added to the upper compartment of a Transwell chamber, and 500 μl of culture medium with 20% FBS was added to the lower compartment. After incubation for 24 h at 37°C, the nonmigrated cells in the upper chamber were gently removed using a wet cotton swab, and the migrated cells were fixed in 4% paraformaldehyde and stained with 0.5% crystal violet. After extensive washing, the stained cells were imaged and counted under a light microscope (×200 magnification; Olympus Corporation, Tokyo, Japan). For the invasion assay, the Transwell filters were precoated with Matrigel® (BD Biosciences). The remaining steps were the same as those used for the migration assay.

### Xenograft tumor model

All experimental procedures involving animals were approved by the Animal Ethics Committee of West China Hospital of Public Health. Short-hairpin RNAs (shRNAs) targeting LINC01124 (sh-LINC01124) and NC shRNA (sh-NC), designed and synthesized by GenePharma, were packaged into a lentivirus. Huh-7 cells were infected with the lentivirus to obtain cells that were stably depleted of LINC01124. Totally 6 nude mice were purchased from Shanghai SLAC Laboratory Animal Company (Shanghai, China) and subcutaneously injected with stable LINC01124-knockdown Huh-7 cells (1 × 10^6^). Each group contained 3 nude mice. The longest (L) and shortest (W) diameters of the tumor xenografts were measured and recorded from days 7 to 30 with a 4-day interval. The tumor volume was calculated using the following formula:

$$Volume=0.5\times L\times W^{2}.$$

At the end of the assay, all mice were euthanized by cervical dislocation, and the tumors were completely excised for further analysis.

### Bioinformatics analysis

ENCORI (https://starbase.sysu.edu.cn/index.php) was used to identify putative miRNAs that directly interacted with LINC01124. The target binding between miR-1247-5p and FOXO3 was predicted using miRDB (http://mirdb.org/) and Targetscan (http://www.targetscan.org).

### Nuclear-cytoplasmic fractionation

The nuclear and cytoplasmic fractions of HCC cells were separated using the Cytoplasmic and Nuclear RNA Purification Kit (Norgen; Thorold, ON, Canada). Following RNA extraction, the relative ratio of LINC01124 in the nuclear and cytoplasmic fractions was determined through quantitative reverse transcriptase-polymerase chain reaction (qRT-PCR). GAPDH and U6 were used as internal controls for the cytoplasmic and nuclear RNA, respectively.

### RNA immunoprecipitation (RIP)

RIP assay was performed to test the binding interaction among LINC01124, miR-1247-5p, and FOXO3 via RNA-induced silencing complexes (RISCs) using the Magna RIP RNA Binding Protein Immunoprecipitation Kit (Merck Millipore, Darmstadt, Germany). Briefly, HCC cell extracts were prepared using RIP lysis buffer, followed by incubation with magnetic beads conjugated with human anti-Ago2 antibody or normal mouse IgG (Merck Millipore). Subsequently, the magnetic beads were collected and incubated with Proteinase K to remove any proteins. The immunoprecipitated RNA was measured by qRT-PCR. IgG acted as a negative control, while input functioned as the positive control.

### Luciferase reporter assay

The regions of LINC01124 and FOXO3 harboring wild-type (wt) miR-1247-5p binding sequences were ligated into the pMIR-luciferase reporter plasmid (Promega, Madison, WI, USA) to produce LINC01124-wt and FOXO3-wt reporter plasmids. Moreover, mutant (mut) segments were created and inserted into the same reporter plasmid, resulting in the formation of LINC01124-mut and FOXO3-mut reporter plasmids. To determine luciferase activity, HCC cells were seeded into 24-well plates and transfected with miR-1247-5p mimic or NC mimic along with wt or mut reporter plasmids using Lipofectamine® 2000. After 2 days, the Dual-Luciferase Reporter Assay (Promega) was used to quantify luciferase activity.

### Western blotting analysis

Cultured cells were resuspended in RIPA buffer (Beyotime, Jiangsu, China) to isolate the total proteins. A bicinchoninic acid protein assay kit (Beyotime) was used to quantify total protein. Equal amounts of protein were separated on 10% SDS–PAGE gels and transferred onto polyvinylidene difluoride membranes. The membranes were blocked with 5% nonfat milk (w/v) for 2 h at room temperature, followed by overnight incubation at 4°C with primary antibodies against FOXO3 (ab109629; dilution 1:800) or GAPDH (ab181602; dilution 1:1000; both from Abcam, Cambridge, MA, USA). Further, horseradish peroxidase-conjugated secondary antibodies (ab205718; dilution 1:5000; Abcam) were added and incubated for 1 h at room temperature. After washing, an enhanced chemiluminescence reagent (Beyotime) was used for signal production. Protein signals were analyzed utilizing Quantity One software version 4.62 (Bio Rad Laboratories, Inc.).

### Statistical analyses

All experiments were repeated for three times, and all results are presented as the mean ± standard deviation. SPSS software 19.0 (SPSS, Chicago, IL, USA) was used to perform statistical analysis. Student’s *t*-test was used for comparisons between two groups, and one-way analysis of variance followed by Tukey’s *post hoc* test was used to evaluate the differences among multiple groups. Statistical significance was set at *p* < 0.05.

## Results

### LINC01124 is overexpressed in HCC and exerts a tumor-promoting role

**Figure 1:**
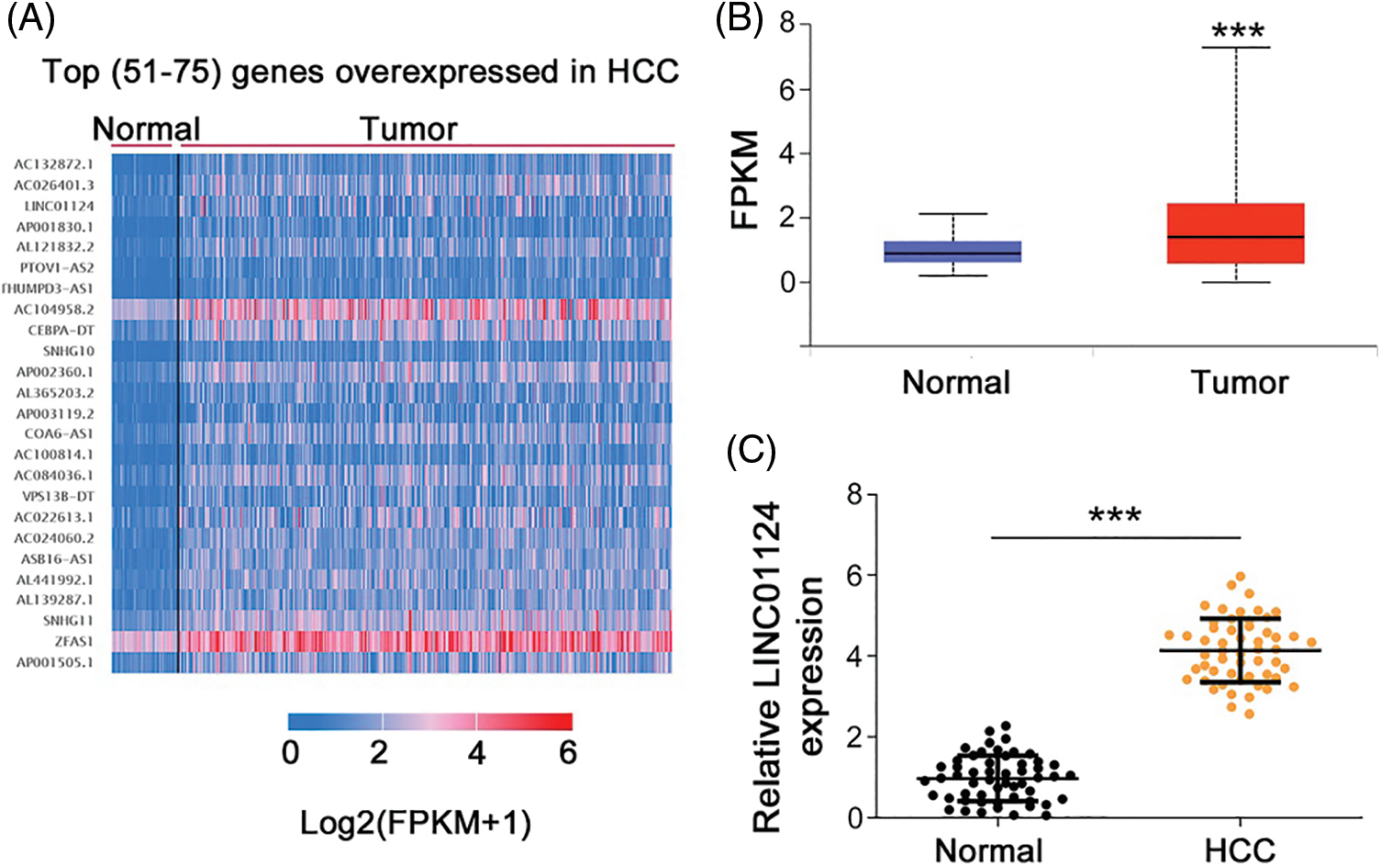
LINC01124 is upregulated in HCC. (A) The top (51-75) genes with overexpressed in HCC. (B) Analysis of LINC01124 expression in LUAD and LUSC from TCGA. (C) qRT-PCR detection of LINC01124 expression in 54 HCC tissues. ***p < 0.001.

We first analyzed the differently expressed lncRNAs in HCC using the Cancer Genome Atlas (TCGA). LINC01124 ranks 53^rd^ among all overexpressed lncRNAs in HCC (Fig. 1A). To thoroughly address the role of LINC01124 in HCC, its expression in HCC was determined using TCGA. Compared with normal liver tissues, HCC tissues show a significant increase in LINC01124 expression (Fig. 1B). To confirm this finding, LINC01124 expression in HCC samples from our own cohort was measured using qRT-PCR, and it was found that LINC01124 was overexpressed in HCC tissues compared with adjacent normal tissues (Fig. 1C).

**Figure 2:**
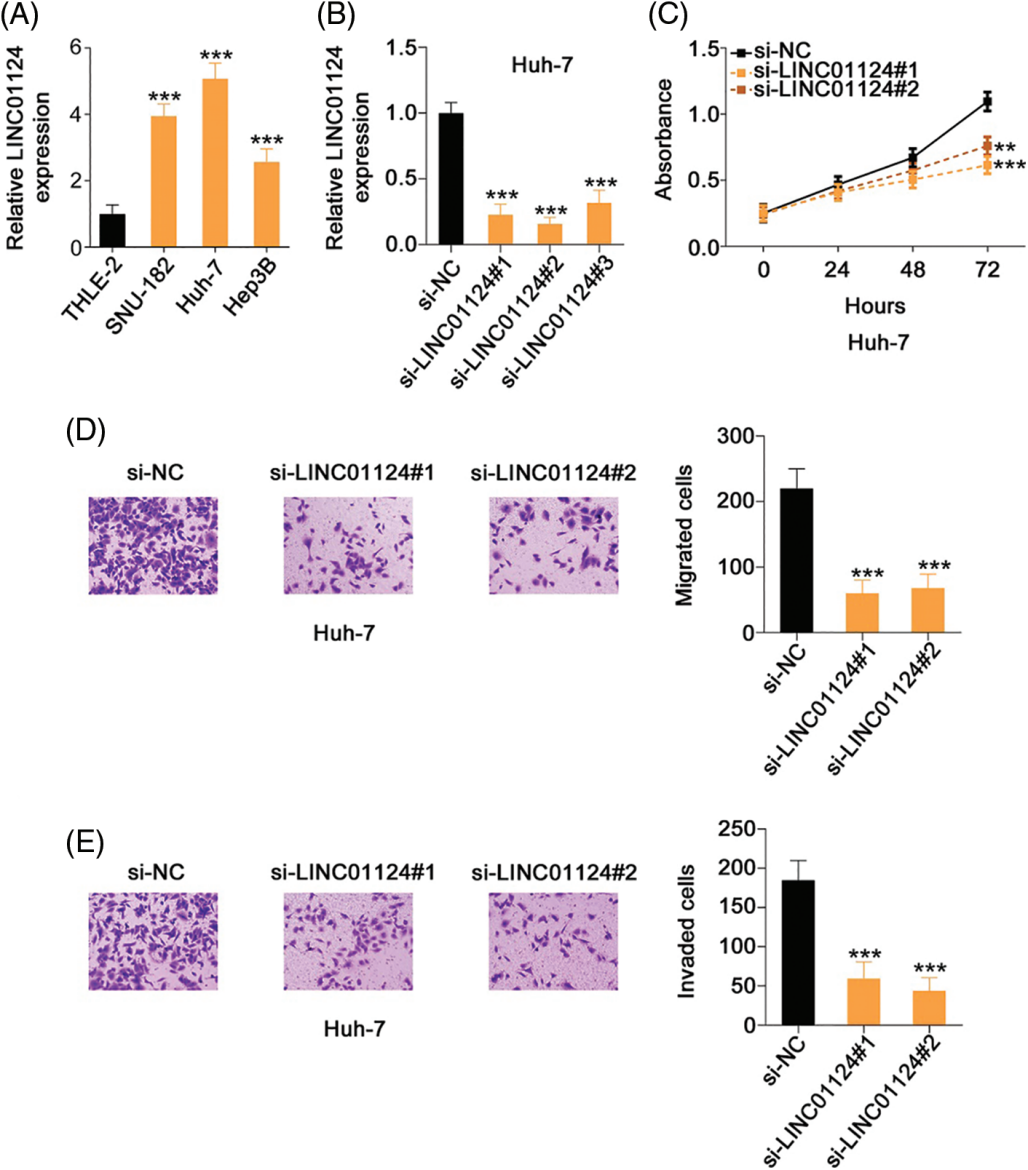
LINC01124 inhibition results in decreased Huh-7 cell proliferation, migration, and invasion *in vitro*. (A) Expression level of LINC01124 in HCC cell lines was detected by qRT-PCR. (B) Transfection efficiency of si-LINC01124 in Huh-7 cells was measured by qRT-PCR. (C) CCK-8 assay was performed to detect the proliferation of Huh-7 cells after si-LINC01124 or si-NC transfection. (D, E) Transwell cell migration and invasion assays assessed the migratory and invasive capacities of Huh-7 cells after LINC01124 knockdown (×200 magnification). **p < 0.01 and ***p < 0.001 (n = 3).

Compared with THLE-2, LINC01124 was highly expressed in the HCC cell lines, particularly in Huh-7 (Fig. 2A). Accordingly, Huh-7 cells were used in the subsequent loss-of-function experiments. Next, si-LINC01124 was used to silence LINC01124 expression in Huh-7 cells (Fig. 2B), and functional experiments were performed to determine whether LINC01124 could regulate HCC behavior. si-LINC01124#1 and si-LINC01124#2 were selected as constructs for the subsequent experiments as they demonstrated the most efficient reduction in LINC01124 expression. Huh-7 cell proliferation decreased concomitantly with LINC01124 knockdown (Fig. 2C). Furthermore, the migratory (Fig. 2D) and invasive (Fig. 2E) capacities of LINC01124-depleted Huh-7 cells were inhibited compared with si-NC-transfected control cells.

**Figure 3:**
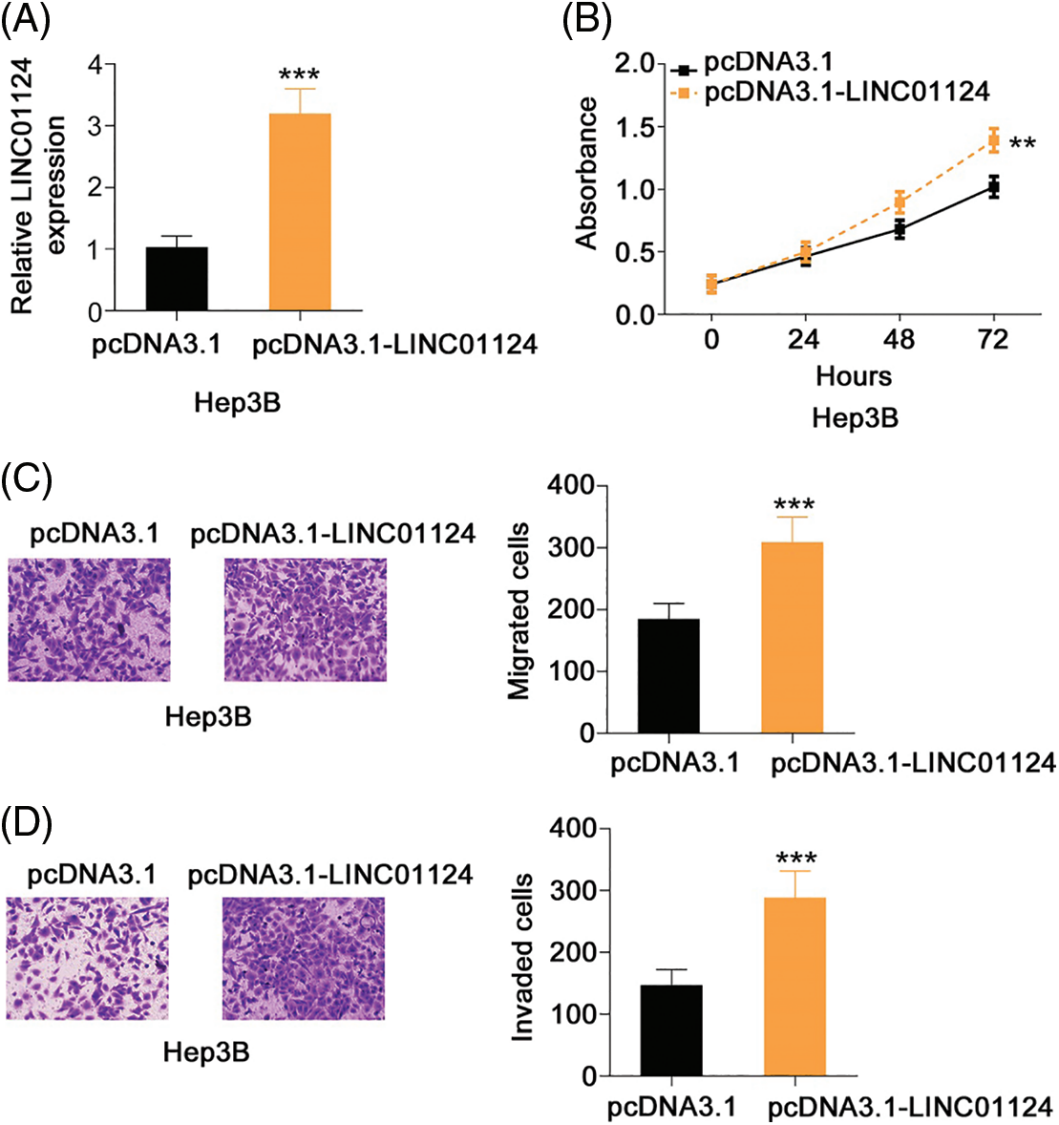
LINC01124 upregulation promotes the aggressiveness of Hep3B cells. (A) LINC01124 level in Hep3B cells after pcDNA3.1-LINC01124 transfection. (B) The proliferation of Hep3B cells after LINC01124 overexpression. (C, D) The motility of Hep3B cells after pcDNA3.1-LINC01124 or pcDNA3.1 treatment (×200 magnification). **p < 0.01 and ***p < 0.001 (n = 3).

We also presented the regulatory effects of LINC01124 overexpression on HCC progression. Hep3B expressed the relative lowest LINC01124 level among all tested HCC cell lines. Therefore, Hep3B was transfected with pcDNA3.1–LINC01124 (Fig. 3A) and used in gain-of-function experiments. Exogenous LINC01124 expression promoted Hep3B cell proliferation (Fig. 3B) and augmented motility (Figs. 3C and 3D). These results suggest that LINC01124 functions as a tumor promoter during HCC progression.

### LINC01124 acts as an miR-1247-5p sponge in HCC cells

**Figure 4:**
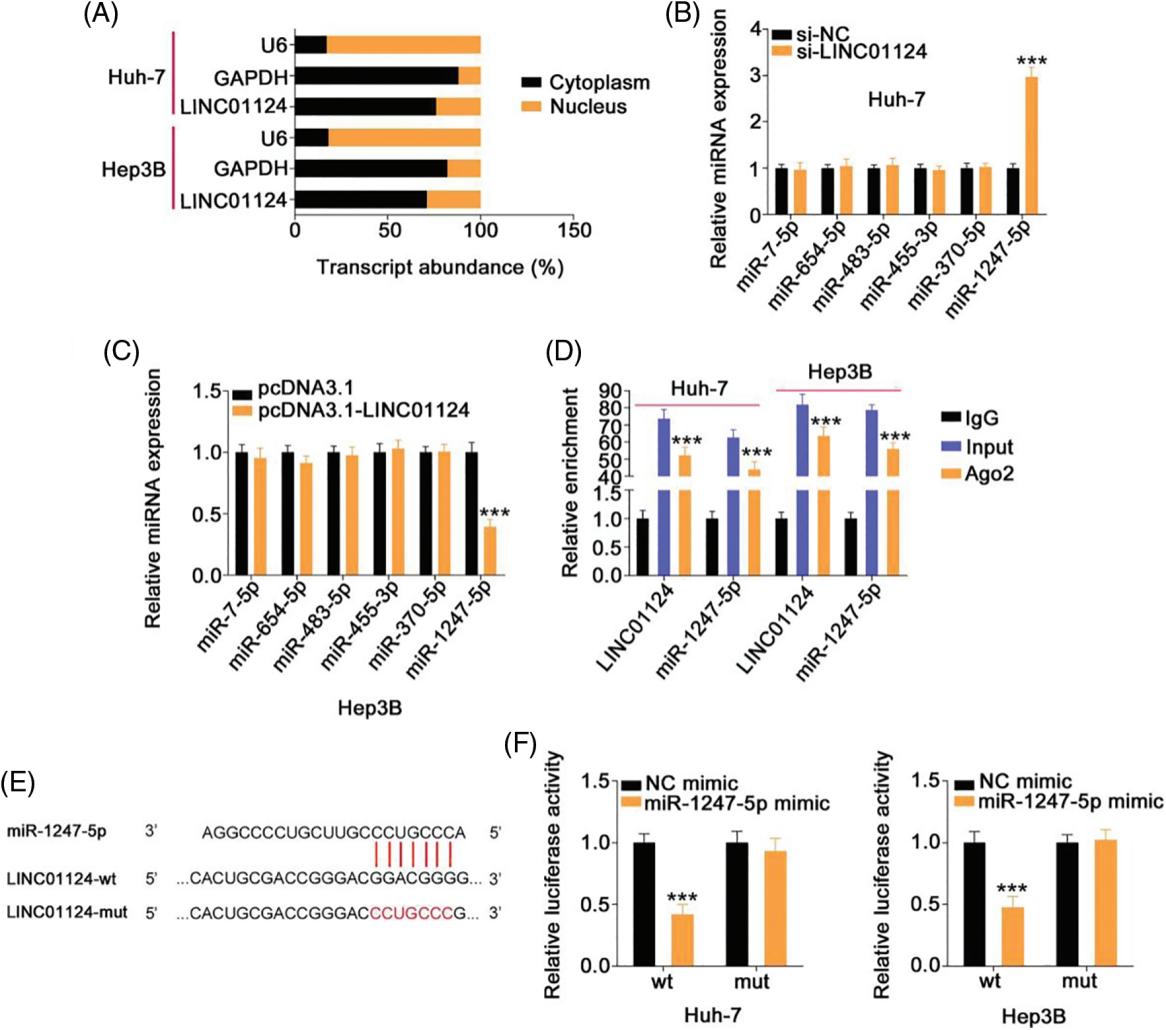
LINC01124 functions by sponging miR-1247-5p in HCC cells. (A) Nuclear-cytoplasmic fractionation assay was done to determine the location of LINC01124 in HCC cells. (B, C) qRT-PCR showed the expression of candidates in HCC cells after LINC01124 deficient or overexpression. (D) RIP assay was carried out using Ago2 antibody and qRT-PCR to measure the enrichment of LINC01124 and miR-1247-5p in HCC cells. (E) The wild-type and mutant binding sequences between LINC01124 and miR-1247-5p were manifested. (F) Luciferase reporter assay evaluated the luciferase activity of HCC cells that were cotransfected with miR-1247-5p mimic or NC mimic and LINC01124-wt or LINC01124-mut. ***p < 0.001 (n = 3).

To determine the molecular mechanism underlying LINC01124, the subcellular localization of LINC01124 was investigated through nuclear-cytoplasmic fractionation. Our results demonstrated that LINC01124 was mostly distributed in the HCC cell cytoplasm (Fig. 4A), suggesting that LINC01124 acts as a ceRNA or miRNA sponge. Using ENCORI, a total of 12 miRNAs containing a potential binding site for LINC01124 were identified. Using the TCGA dataset, we identified six downregulated miRNAs in HCC, including miR-7-5p, miR-654-5p, miR-483-5p, miR-455-3p, miR-370-5p, and miR-1247-5p. Therefore, these miRNAs were subjected to subsequent experimental certification.

To determine the regulatory effect of LINC01124 on miRNA expression, qRT-PCR was conducted to measure miRNA expression on LINC01124-silenced or LINC01124-overexpressed HCC cells. Interference with LINC01124 resulted in miR-1247-5p upregulation in Huh-7 cells, whereas miR-1247-5p decreased in Hep3B cells in response to LINC01124 upregulation (Figs. 4B and 4C). RIP assays were used to further verify the interaction between LINC01124 and miR-1247-5p in the HCC cells. As shown in Fig. 4D, LINC01124 and miR-1247-5p were both enriched by the Ago2 antibody in HCC cells, implying that these two molecules were recruited to the RNA-induced silencing complex for potential functional interactions. Additionally, the direct binding of LINC01124 and miR-1247-5p (Fig. 4E) was confirmed in the luciferase reporter assay. The luciferase activity of LINC01124-wt decreased with miR-1247-5p overexpression, whereas that of LINC01124-mut remained unchanged after miR-1247-5p mimic cotransfection (Fig. 4F). These data collectively indicate that LINC01124 functions as a miR-1247-5p sponge in HCC cells.

### Regulatory activities of LINC01124 on HCC cells dependent on miR-1247-5p

**Figure 5:**
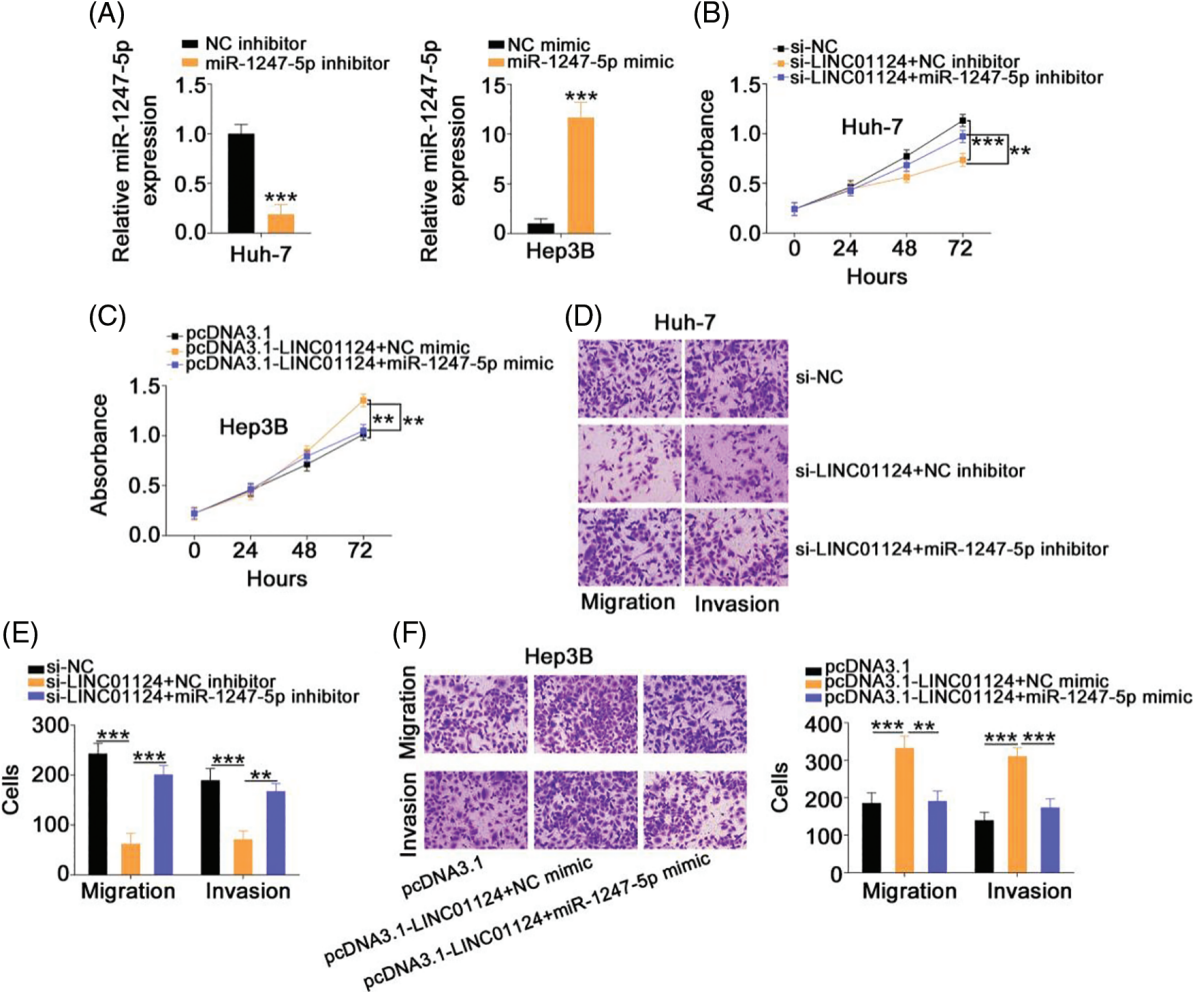
LINC01124 exerts its tumor-promoting roles in HCC cells via regulating miR-1247-5p. (A) The efficiency of miR-1247-5p inhibitor in Huh-7 cells and miR-1247-5p mimic in Hep3B cells. (B, C) MiR-1247-5p inhibitor or NC inhibitor was transfected into LINC01124-depleted Huh-7 cells. MiR-1247-5p mimic or NC mimic was transfected into LINC01124-overexpressed Hep3B cells. After transfection, cell proliferation was detected by CCK-8 assay. (D-F) Transwell cell migration and invasion assays were applied to evaluate the migration and invasion of HCC cells treated as above described (×200 magnification) **p < 0.01 and ***p < 0.001 (n = 3).

Rescue experiments were designed to understand whether LINC01124 facilitates HCC progression by regulating miR-1247-5p. A significant decrease in miR-1247-5p expression was found in the miR-1247-5p inhibitor treatment Huh-7 cells. qRT-PCR also confirmed that miR-1247-5p was significantly overexpressed in miR-1247-5p mimic-transfected Hep3B cells (Fig. 5A). Functional experiments revealed the inhibition of Huh-7 cell proliferation (Fig. 5B) upon LINC01124 silencing, which was restored by inhibiting miR-1247-5p expression. Furthermore, we observed that the Hep3B cell proliferation promoted by LINC01124 overexpression was recovered when miR-1247-5p was re-expressed (Fig. 5C). Additionally, the migratory and invasive capacities of Huh-7 cells impaired by LINC01124 downregulation were offset by miR-1247-5p inhibitor treatment (Figs. 5D and 5E). Moreover, the effect of LINC01124 upregulation on Hep3B cell motility was counteracted by miR-1247-5p mimic cotransfection (Fig. 5F). Taken together, the pro-oncogenic actions of LINC01124 on HCC cells can be attributed to miR-1247-5p.

### FOXO3 is the downstream target of miR-1247-5p in HCC cells

**Figure 6:**
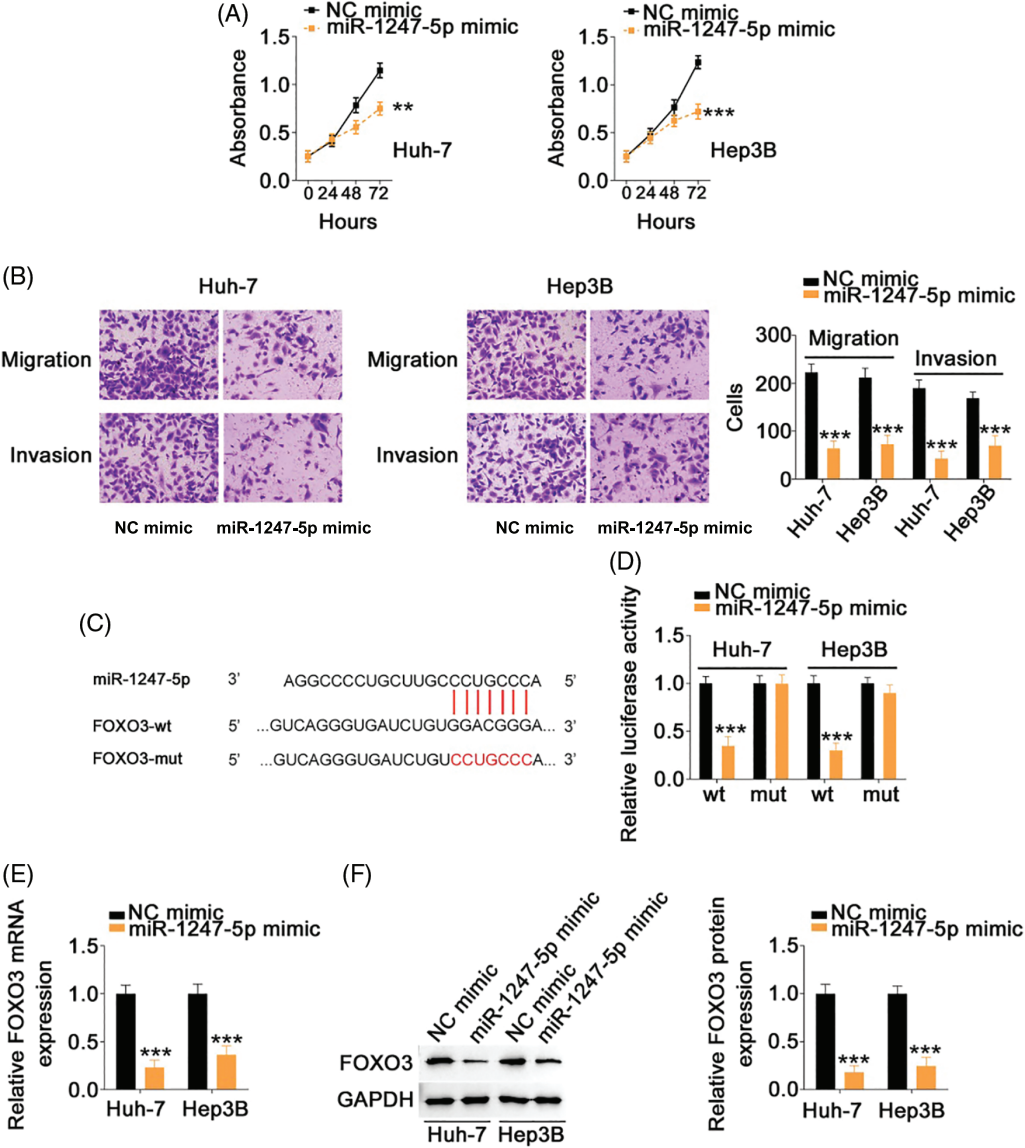
MiR-1247-5p directly targets FOXO3 in HCC cells. (A) CCK-8 assay was used to evaluate the proliferation of miR-1247-5p-overexpressing HCC cells. (B) The migration and invasion of HCC cells with miR-1247-5p mimic or NC mimic transfection was determined by Transwell assays (×200 magnification). (C) The wild-type and mutant binding sites of miR-1247-5p within FOXO3 3’-UTR. (D) Luciferase activity of FOXO3-wt or FOXO3-mut was detected in HCC cells after co-transfection with miR-1247-5p mimic or NC mimic. (E, F) qRT-PCR and western blot analysis were used to detect FOXO3 mRNA and protein levels, respectively, in miR-1247-5p-overexpressed HCC cells **p < 0.01 and ***p < 0.001 (n = 3).

The specific role of miR-1247-5p in HCC cells was also revealed in detail. The miR-1247-5p mimic and NC mimic were transfected into HCC cells. Functional experiments indicated that transfection with the miR-1247-5p mimic resulted in a decrease in cell proliferation (Fig. 6A) in HCC cells. Additionally, overexpression of miR-1247-5p inhibited the migration and invasion (Fig. 6B) of HCC cells.

Using bioinformatics analysis, a potential binding site of miR-1247-5p was identified in FOXO3 3’-UTR (Fig. 6C). To determine whether miR-1247-5p directly targets FOXO3 in HCC cells, a luciferase reporter assay was implemented in HCC cells after transfection with miR-1247-5p mimic or NC mimic together with FOXO3-wt or FOXO3-mut. Ectopic miR-1247-5p expression significantly reduced the luciferase activity of HCC cells transfected with FOXO3-wt, whereas the luciferase activity of FOXO3-mut exhibited no change in response to ectopic miR-1247-5p expression (Fig. 6D). Furthermore, enforced miR-1247-5p expression reduced FOXO3 expression in HCC cells (Figs. 6E and 6F). The above data identified FOXO3 as a downstream target of miR-1247-5p in HCC cells.

### FOXO3 contributes to the cancer-promoting roles of LINC01124 in HCC cells

**Figure 7:**
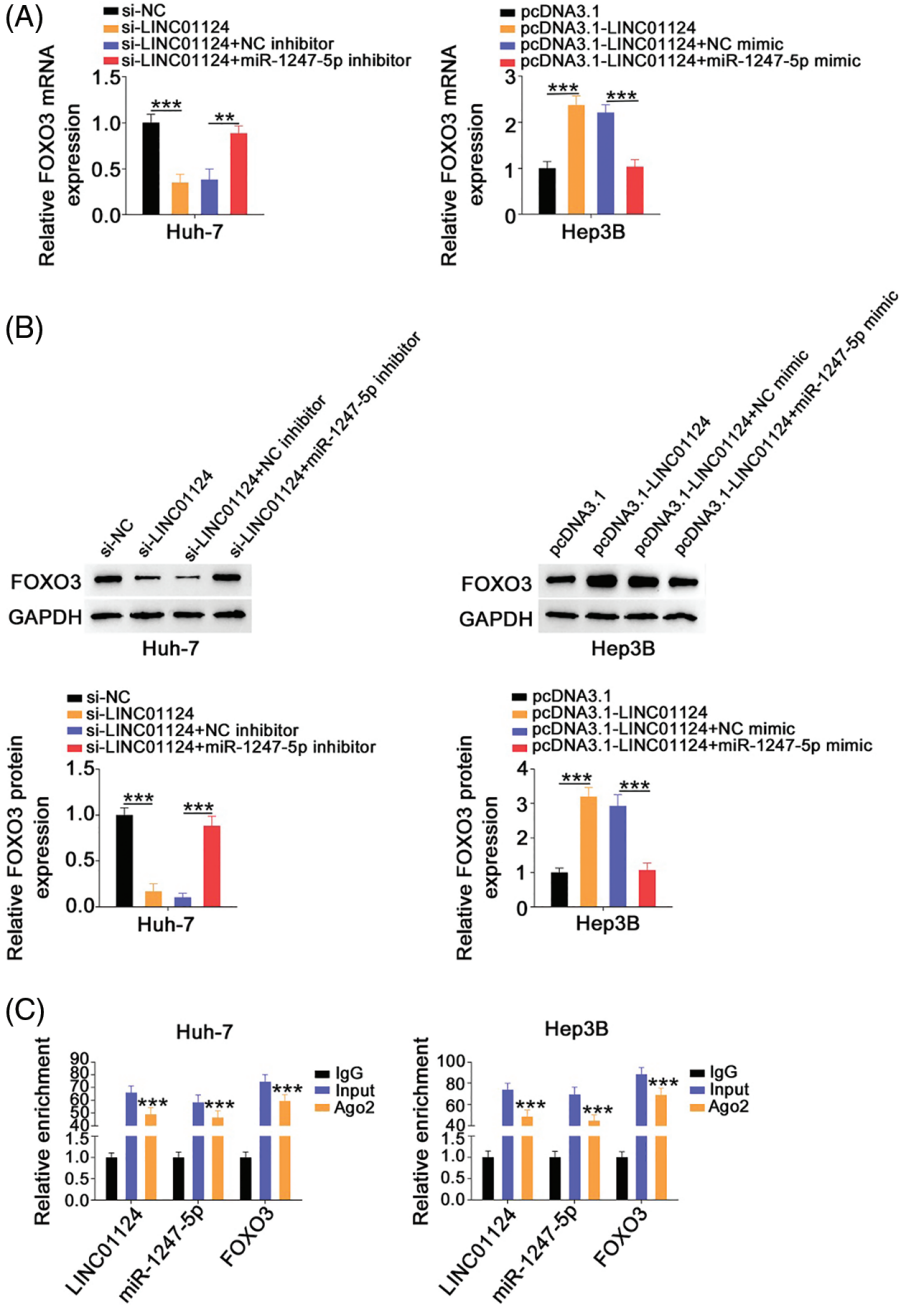
LINC01124 activates FOXO3 expression in HCC cells by decoying miR-1247-5p. (A, B) Huh-7 cells were transfected with si-NC, si-LINC01124, si-LINC01124+NC inhibitor or si-LINC01124+miR-1247-5p inhibitor. Hep3B cells were transfected with pcDNA3.1, pcDNA3.1-LINC01124, pcDNA3.1-LINC01124+NC mimic or pcDNA3.1-LINC01124+miR-1247-5p mimic. (C) RIP assay was performed using Ago2 antibody and qRT-PCR was used to measure the enrichment of LINC01124, miR-1247-5p, and FOXO3 in HCC cells. **p < 0.01 and ***p < 0.001 (n = 3).

After revealing FOXO3 as a direct target of miR-1247-5p in HCC, we next examined whether LINC01124 affects FOXO3 expression in HCC cells and investigated the underlying mechanism. FOXO3 levels were decreased in Huh-7 cells following LINC01124 depletion, whereas miR-1247-5p inhibitor cotransfection reversed this effect (Figs. 7A and 7B). Furthermore, reintroduction of LINC01124 induced a signification upregulation of FOXO3 levels in Hep3B cells, and the regulatory action was reversed by treatment of miR-1247-5p mimic (Figs. 7A and 7B). In addition, RIP assays demonstrated that LINC01124, miR-1247-5p, and FOXO3 were all enriched following immunoprecipitation with Ago2 antibody in HCC cells (Fig. 7C). In short, FOXO3 is under the control of LINC01124 through sequestration of miR-1247-5p.

**Figure 8:**
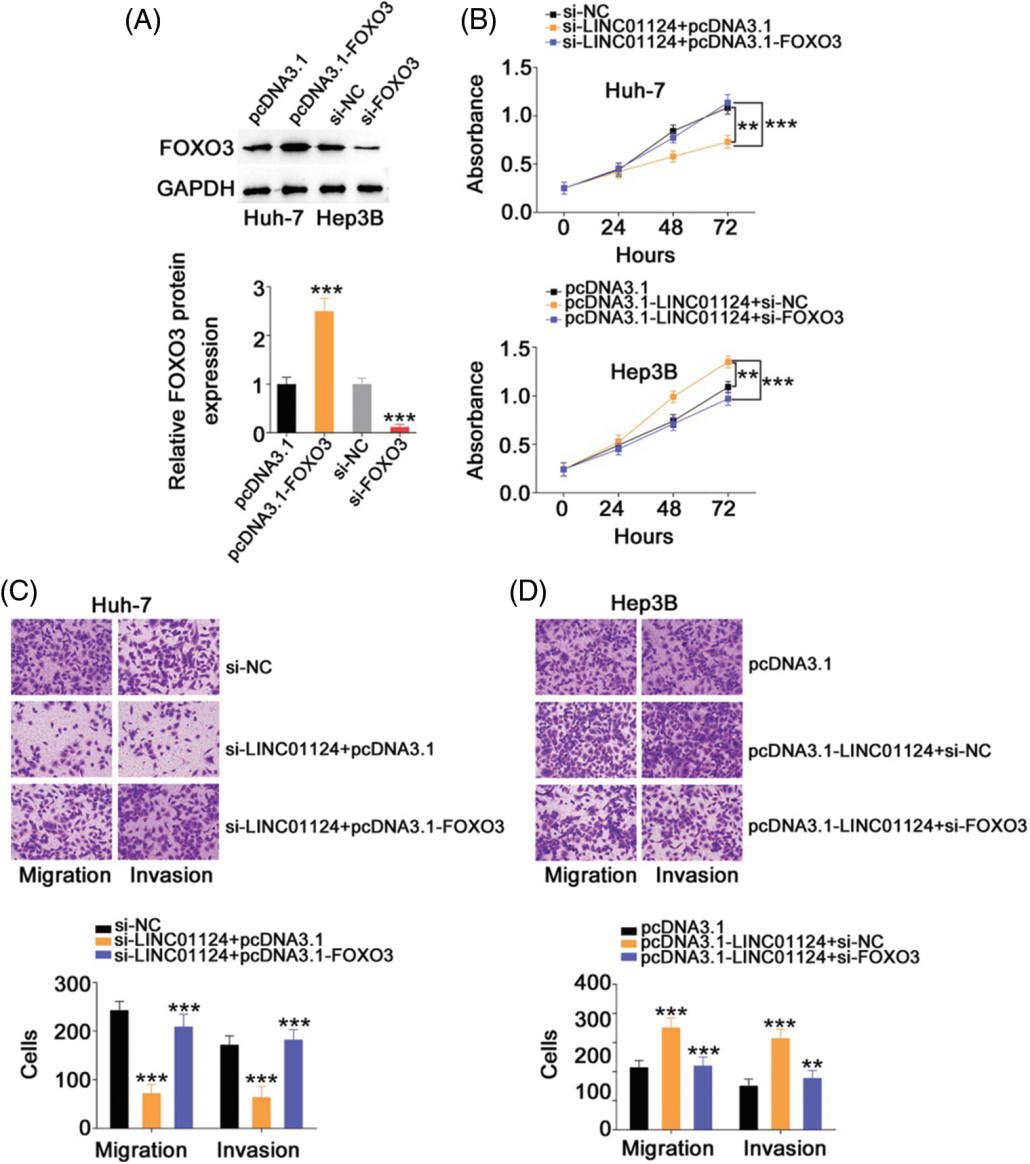
Downregulation of FOXO3 reverses the effects in HCC cells induced by LINC01124 upregulation. (A) Western blotting analysis detected the FOXO3 protein level in HCC cells that were transfected with pc-FOXO3 or si-FOXO3. (B-D) The pc-FOXO3 or pcDNA3.1 plasmid in combination with si-LINC01124 was introduced into Huh-7 cells. Hep3B cells were transfected with pcDNA3.1, pcDNA3.1-LINC01124+si-NC, or pcDNA3.1-LINC01124+si-FOXO3. The proliferation, migration (×200 magnification), and invasion (×200 magnification) were determined by CCK-8 assay, and Transwell cell migration assay and invasion assay, respectively. **p < 0.01 and ***p < 0.001 (n = 3).

Rescue experiments were also realized to address whether FOXO3 is a key indirect target of LINC01124 in HCC. The si-FOXO3 and FOXO3-overexpressing plasmid pcDNA3.1-FOXO3 were used in rescue experiments, and their transfection efficiency was demonstrated by western blot analysis (Fig. 8A). Interference with LINC01124 resulted in decreased proliferation, migration and invasion (Figs. 8B and 8C) of Huh-7 cells; nevertheless, these regulatory effects were abolished by FOXO3 upregulation. Similarly, cotransfection of si-FOXO3 was capable to abrogate the effects of pcDNA3.1-LINC01124 on Hep3B cell proliferation (Fig. 8B) and motility (Fig. 8D). Overall, the miR-1247-5p/FOXO3 axis contributes to the tumor-promoting activities of LINC01124 in HCC cells.

### Downregulation of LINC01124 suppresses HCC tumor growth in vivo

**Figure 9:**
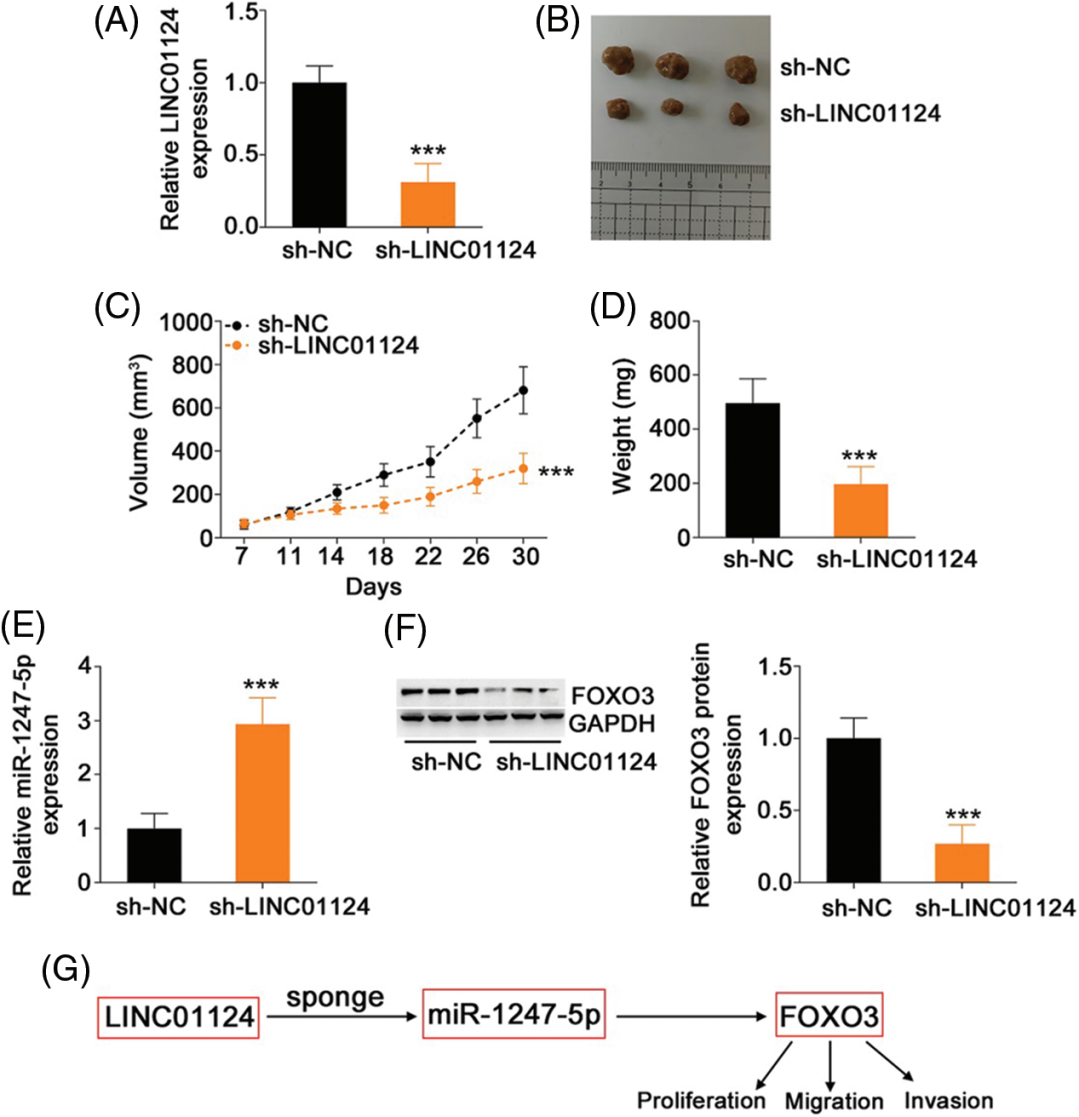
Loss of LINC01124 restricts tumor growth of HCC cells *in vivo*. (A) Lentivirus carrying sh-LINC01124 or sh-NC was transduced into Huh-7 cells. The efficiency in silencing LINC01124 expression was confirmed by qRT-PCR. (B) Representative image of the tumor xenografts obtained from the sh-LINC01124 and sh-NC groups. (C) Tumor size was recorded from day 7 to day 30 with a 4-day interval. The resulting data was used for plotting the growth curve. (D) All mice were euthanized at the end of this assay, and tumor xenografts were weighed. (E) Expression of miR-1247-5p in tumor xenografts was measured by qRT-PCR. (F) Western blot analysis was used to measure FOXO3 protein level in the tumor xenografts. (G) miR-1247-5p–FOXO3 axis was situated downstream of LINC01124 in HCC. ***p < 0.001 (n = 3).

A xenograft tumor model was established to assess the impact of LINC01124 on HCC tumor growth *in vivo*. Huh-7 cells were transduced with lentivirus carrying sh-LINC01124 or sh-NC, and the cells were subcutaneously injected into nude mice. The transfection efficiency of sh-LINC01124 was measured by qRT-PCR. Transfection of sh-LINC01124 significantly decreased LINC01124 expression in Huh-7 cells (Fig. 9A). The tumor xenografts obtained from the sh-LINC01124 group exhibited reduced volume (Figs. 9B and 9C) and weight (Fig. 9D) compared with those in the sh-NC group. In addition, tumor xenografts derived from Huh-7 cells with stable LINC01124 silencing featured increased miR-1247-5p (Fig. 9E) levels in contrast to tumors in the sh-NC group. Furthermore, FOXO3 protein level was downregulated in the tumor xenografts with stable LINC01124 knockdown (Fig. 9F). Overall, LINC01124 depletion attenuated the growth of HCC cells *in vivo*.

## Discussion

In recent decades, several studies have identified and described the presence of dysregulated lncRNAs in HCC cells [27–29]. LncRNAs are transcriptional/translational regulators that are considered as an important driving force underlying HCC etiology and progression [30]. Although genomic studies have identified over 50,000 lncRNAs in the human genome [31], the function of most lncRNAs has not been explored in HCC. Consequently, the primary goal of our study was to elucidate the role of LINC01124 in the aggressiveness of HCC cells and to identify the underlying regulatory mechanism. Our findings may provide significant insight for the identification of effective diagnostic biomarkers and therapeutic targets for HCC.

LINC01124 is a weakly-expressed RNA gene in non-small cell lung cancer that exhibits a distinct relationship with the patients' age and distant metastasis [26]. Functionally, LINC01124 is confirmed as an anti-oncogenic lncRNA in non-small cell lung cancer that has been found to and contribute to the regulation of many aggressive phenotypes [26]. Nevertheless, the expression and functions of LINC01124 in HCC remain undefined. In this study, LINC01124 expression was found to be elevated in HCC tissues and cell lines. Depletion of LINC01124 decreased cell proliferation, migration, and invasion, whereas ectopic LINC01124 expression resulted in the opposite results. Additionally, ablation of LINC01124 resulted in tumor growth impairment *in vivo*. Thus, LINC01124 tissue specificity was confirmed in its expression profile and specific functions in human cancers.

The ceRNA theory has recently been proposed as a novel mechanism of gene regulation [32]. This theory postulates that lncRNAs are primarily located in the cytoplasm and function as miRNA sponges to protect miRNA-targeted mRNAs from degradation, thereby regulating gene expression and affecting the malignancy of human cancers [33,34]. The downstream molecular events associated with LINC01124 in regulating the oncogenicity of HCC were comprehensively studied in this study. Nuclear-cytoplasmic fractionation experiments identified LINC01124 as a cytoplasmic lncRNA in HCC cells, suggesting that LINC01124 functions as a ceRNA to decoy its target miRNAs. Bioinformatics analysis was performed, and a potential miR-1247-5p binding site was identified within LINC01124. Further studies revealed that LINC01124 interference results in a significant increase in miR-1247-5p expression in Huh-7 cells, whereas LINC01124 overexpression downregulates miR-1247-5p in Hep3B cells. Subsequent luciferase reporter and RIP assays revealed a direct crosslink between miR-1247-5p and LINC01124 in HCC cells. Collectively, our results indicate that LINC01124 functions as a ceRNA in HCC cells by sequestering miR-1247-5p.

MiR-1247-5p is downregulated in several types of human cancer [35–38]. In agreement with a previous study [39], miR-1247-5p was weakly expressed in HCC and exerted cancer-inhibiting activities through affecting malignant properties. The results of our mechanistic experiments demonstrated that FOXO3 was a direct target of miR-1247-5p. Additional in-depth studies were performed to establish the relationship among LINC01124, miR-1247-5p, and FOXO3 in HCC. Molecular analysis was performed and showed that FOXO3 expression was positively regulated by LINC01124 in HCC cells, whereas governing miR-1247-5p expression reversed the regulatory actions of LINC01124 on FOXO3 expression. More importantly, LINC01124, miR-1247-5p, and FOXO3 were collectively recruited to RISCs in HCC cells. Altogether, the interactions among LINC01124, miR-1247-5p, and FOXO3 constitute a novel ceRNA pathway in HCC which may serve as a new treatment target in the field of HCC management.

As a member of the Forkhead box class O transcription factor family, highly expressed FOXO3 in HCC executes tumor-promoting activities during oncogenesis and progression, and it is involved in various tumor processes [40–42]. Interestingly, this study revealed that FOXO3 was negatively regulated by miR-1247-5p and positively regulated by LINC01124 in HCC cells. Finally, rescue assays revealed miR-1247-5p–FOXO3 axis output inhibition was sufficient to counteract changes in the malignant process caused by LINC01124. All these results highlight that the miR-1247-5p–FOXO3 axis is situated downstream of LINC01124 in HCC.

At present, onco-lncRNA targeted therapy is a novel and promising therapeutic method for patients with cancer [43–45]. Despite their low abundance, the functional diversity of lncRNAs renders these noncoding RNA molecules promising therapeutic targets because they can exert extensive effects on cellular programs and phenotypes [46]. Moreover, HCC remains an incurable and fatal human malignancy because the efficacy of current primary treatment modalities remains very low. Therefore, our investigation of LINC01124 in HCC may improve our understanding of the disease progression and may be conducive in facilitating novel anticancer treatment strategies in what has been a losing battle up until now.

Our research contained several limitations. Firstly, we did not explore the relationship between LINC01124 and prognosis of patients with HCC. Secondly, the regulatory effect of LINC01124 on the metastasis of HCC cells *in vivo* was not examined. Lastly, the exact mechanisms underlying the dysregulation of LINC01124 in HCC remain unclear. We will resolve these limitations in the following experiments.

To conclude, our results offer solid evidence regarding the pro-oncogenic role of LINC01124 in upregulating FOXO3 in HCC by sequestering miR-1247-5p (Fig. 9G). The proposed mechanism of the miR-1247-5p–FOXO3 axis may be helpful for better understanding the role of LINC01124 in HCC oncogenesis and promoting the identification of promising therapeutic strategies for cancer treatment.

## Data Availability

The data of the current study is available from the corresponding author on reasonable request.
